# Adsorption Studies of *Salmonella* Enteritidis and *Escherichia coli* on Chitosan-Coated Magnetic Nanoparticles

**DOI:** 10.3390/cells14030225

**Published:** 2025-02-05

**Authors:** Anthony James Franco, Evangelyn Alocilja

**Affiliations:** 1 Department of Biosystems and Agricultural Engineering, Michigan State University, East Lansing, MI 48824, USA; francoa5@msu.edu; 2 Global Alliance for Rapid Diagnostics, Michigan State University, East Lansing, MI 48824, USA

**Keywords:** magnetic nanoparticles, bacterial adsorption, *Salmonella*, *E. coli*

## Abstract

One of the challenges of microbiological testing is the complex and lengthy sample preparation, causing delays in getting the final result. Immunomagnetic separation is one of the sample preparation techniques recently used to overcome this complexity. However, it is expensive, fragile, and requires cold storage. This study aimed to use chitosan-coated magnetic nanoparticles (cMNP) to capture bacterial cells from a simulated matrix and understand the interaction between the bacteria and the cMNP using batch adsorption studies. To illustrate the concept, *Salmonella* Enteritidis and *Escherichia coli* were used. Results showed that the adsorption of *Salmonella* Enteritidis and *E. coli* fitted the pseudo-second-order kinetic model (R^2^ = 0.939 and 0.968, respectively) and the Freundlich isotherm model (R^2^ = 0.999 and 0.970, respectively). The increased ionic strength enhanced bacterial adsorption, and the highest capture efficiency was observed at pH 4 (32.8% and 98.1% for *Salmonella* Enteritidis and *E. coli*, respectively). These results show that chemisorption plays a significant role in bacterial adsorption to cMNP. Furthermore, increasing ionic strength and acidic pH (pH 4) significantly affects the adsorption of *Salmonella* Enteritidis and *E. coli* on cMNP, making them crucial for enhancing the performance of cMNP-based sample preparation methods.

## 1. Introduction

Magnetic nanoparticles (MNP) are becoming increasingly popular in detection systems because of their low production cost, stability and biocompatibility, and environment-friendly nature [1,2,3]. Another advantage of using them in detection systems is that their surfaces can be easily modified with various inorganic and organic compounds, polymers, antibodies, proteins, enzymes, nucleotides, and carbohydrates [1,3,4]. Surface modification of MNP allowed for more robust MNP-based nano-biosensors and sample preparation systems [4]. Modifying MNP with gold made it a promising material for bioseparation and electrochemical and optical sensing [5]. MNP coated with polymeric and carbon-based materials stabilized MNP and allowed for more efficient conjugation of antibodies to be used in immunoassays [6]. Polyaniline coating facilitated the conjugation of the MNP with oligonucleotides and made the MNP electroactive to be used for the detection of DNA targets [7,8].

Chitosan is one of the materials used to modify the surface of MNP to introduce chemical functionalities [9,10,11,12,13]. It is a polysaccharide composed of D-glucosamine and N-acetyl D-glucosamine units [14]. Some of its biologically important physicochemical properties, such as solubility and antimicrobial activity, depend on pH, its degree of deacetylation, and molecular weight [14,15]. Despite its antimicrobial properties, chitosan-coated MNP (cMNP) has been reported to capture various bacteria effectively, which were then detected using culture-dependent and culture-independent methods [13,16,17,18,19]. The successful bacterial capture can be attributed to the interaction between the bacterial target and the MNP. It was proposed that specific interactions between various bacterial surface proteins and the chitosan coating of the MNP resulted in the formation of a stable conjugate that could easily be recovered using a simple magnet [20]. This sample preparation method can be effective for simplifying and speeding up the analysis of samples for important foodborne pathogens, such as *Salmonella* Enteritidis and *E. coli*. Understanding the mechanisms and factors involved in the adsorption of these bacteria on cMNP is crucial to improving this technique.

The cMNP has shown promise in extracting bacteria from complex matrices [13,16,17,18,21,22,23]. However, these studies have only hypothesized the nature of interaction. For the first time, this paper explored the interaction between the bacteria (*Salmonella* Enteritidis and *E. coli*) and the cMNP using adsorption kinetics and isotherm models, including the effects of ionic strength and pH. This is also the first study examining these models at a low cell concentration range of 10^3^–10^5^ CFU/mL. The long-term goal is to develop a rapid, cMNP-based sample preparation procedure suitable for simultaneous multi-target detection.

## 2. Materials and Methods

### 2.1. Materials

Proprietary chitosan-coated MNP was synthesized in-house at the Nano-Biosensors Laboratory [24]. The cMNP is composed of a paramagnetic iron oxide core and a chitosan shell. The paramagnetic core was synthesized from ferric chloride hexahydrate (FeCl_3_·6H_2_O) precursor using ethylene glycol as a reducing agent and sodium acetate as a porogen. The core was coated by polymerizing chitosan. Reagents used in the synthesis were purchased from Sigma-Aldrich (Burlington, MA, USA). The cMNP suspension used throughout this study was prepared by suspending the cMNP in sterile type 1 water at a final concentration of 5 mg/mL. The cMNP suspension was sonicated for 60 min, stored in the dark at room temperature when not in use, and sonicated for 15 min before every experiment to disperse completely in the suspension. Phosphate-buffered saline, pH 7.4 (PBS) premix was purchased from VWR International (Radnor, PA, USA), dehydrated tryptic soy agar (TSA) and tryptic soy broth (TSB) were purchased from MilliporeSigma (Burlington, MA, USA), and they were prepared as recommended by the supplier. HCl and NaOH solutions (0.1 M) were prepared. Magnetic racks were purchased from Promega Corporation (Madison, WI, USA). Formvar/carbon-supported copper grids (formvar/carbon 200 mesh copper) used for the transmission electron microscope (TEM) imaging were purchased from Electron Microscopy Systems (Hatfield, PA, USA). Glutaraldehyde, cacodylate buffer, and uranyl acetate stain were provided by the Center for Advanced Microscopy, Michigan State University.

### 2.2. Bacterial Cultures

Cultures of *Salmonella* Enteritidis ATCC BAA-1045 and *Escherichia coli* ATCC 15597 were used in the experiments. Stock bacterial suspensions were prepared by transferring a single colony of the bacteria from an agar culture to 9 mL TSB and allowing it to grow for 18–24 h at 37 °C. From the stock bacterial suspension, a 1 mL aliquot was transferred to 9 mL TSB, and the resulting suspension was incubated at 37 °C for 3 to 4 h. All experiments used bacterial cultures grown for 3–4 h.

### 2.3. Transmission Electron Microscopy

The cMNP, bacteria, and their conjugates were visualized using transmission electron microscopy (JEM-1400 Flash, Jeol, Nieuw-Vennep, Tokyo, Japan) in the Center for Advanced Microscopy, Michigan State University. The conjugates were fixed by glutaraldehyde fixation. The cMNP–bacteria conjugates were pelleted first by placing the tube in a magnetic rack for 1 min and then fixed using 2.5% glutaraldehyde in 0.1 M cacodylate buffer. The fixed cells were washed twice and resuspended using 0.1 M cacodylate buffer. The cMNP suspension was used without additional preparation. After the preparation, 10 µL aliquots were placed in formvar-coated copper grids and incubated for 10 min. The grids were carefully blot-dried and stained with 0.1% uranyl acetate.

### 2.4. Adsorption Kinetics and Isotherm Experiments

The experiments were performed at pH 7.4 using PBS. A 100 µL aliquot of the 5 mg/mL cMNP suspension was added into 1 mL of the buffer containing bacterial cells. The mixture was mixed gently and allowed to stand for a certain incubation period. After incubation, the mixture was magnetically separated for 1 min. The supernatant was recovered, and the residue was resuspended with 1 mL of the buffer. The residue contains the formed cMNP–bacteria conjugate. The recovered supernatant and the resuspended pellet were plated in TSA, and the colonies were counted. Based on the colony counts, the adsorption capacity was calculated (Equation (1); Supplementary Material S2).

(1) $$Adsorption\;capacity=\frac{cells\;adsorbed}{mass\;of\;MNP\;used}\;.$$

Adsorption kinetics experiments were carried out at different incubation periods (i.e., 5, 10, 15, 30, and 60 min) at a cell concentration of 10^4^ CFU/mL. The adsorption capacity was calculated, plotted against the incubation time, and fitted to the non-linear forms of the pseudo-first-order and pseudo-second-order kinetic models (Table 1).

Adsorption isotherm experiments were performed at different cell concentrations (ranging from 10^3^ to 10^5^ CFU/mL) and incubated for 60 min. The adsorption capacity was calculated, plotted against the cell concentration of the supernatant, and fitted to Langmuir and Freundlich isotherms (Table 2). The Langmuir and the Freundlich isotherms were used to model the adsorption equilibrium of bacteria on surfaces [28,29,30,31]. Both adsorption kinetics and isotherm experiments were performed with three technical replicates (n = 3).

### 2.5. Point of Zero Charge (PZC) Determination

The PZC of the cMNP was determined by the salt addition method [34]. A 1 mL aliquot of the cMNP suspension in Type 1 water (5 mg/mL) was added to 9.0 mL of 0.1 M NaCl solution in a 50 mL centrifuge tube. The pH of the mixture was adjusted using 0.1 M HCl or NaOH solutions to obtain a pH of 2, 3, 4, 5, 6, and 7. These initial pH values were denoted as pH_i_. The resulting mixture was shaken for 24 h, after which the pH was measured (pH_f_). The difference in pH (ΔpH = pH_f_ − pH_i_) was plotted against pH_i_. The PZC was determined from this plot by determining the pH at which the ΔpH intersects the x-axis. The experiment was performed with two technical replicates (n = 2).

### 2.6. Zeta Potential Measurement

The cMNP, *Salmonella* Enteritidis, and *E. coli* suspensions were prepared by dilution using a sterile dispersant. The cMNP was diluted to a final concentration of 0.05 mg cMNP/mL, and the bacterial suspensions to 10^6^ CFU/mL. After dilution, samples were placed in a Folded Capillary Zeta Cell (DTS1070, Malvern Pananalytical Ltd., Malvern, UK), and then the zeta potential was measured using Zetasizer Nano ZS (Malvern Pananalytical Ltd., Malvern, UK). The experiment was performed with three technical replicates (n = 3).

### 2.7. Effect of Ionic Strength and pH on Adsorption

The effect of ionic strength was investigated by performing the adsorption experiment using phosphate buffer and phosphate-buffered saline. McIlvaine buffer with different pH values (4, 5, 6, and 7) was used for the effect of pH. The cell concentration was kept at 10^4^ CFU/mL and was incubated with the cMNP for 60 min. The capture efficiency (Equation (2); Supplementary Material S2) for each treatment was calculated based on the colony counts of the residue and the supernatant. The experiments were performed with three technical replicates (n = 3).

(2) $$\begin{array}{c} Capture\;\\ efficiency \end{array}=\frac{cells\;adsorbed}{cells\;adsorbed+cells\;remaining\;on\;the\;supernatant}\times 100.$$

### 2.8. Curve Fitting and Statistical Analyses

Non-linear curve fitting to kinetic and isotherm models was performed using the *nlinfit* built-in function in MATLAB^®^ version R2023b. Statistical tests (i.e., Welch’s test and analysis of variance followed by Tukey’s honest significant difference (HSD) test) were performed using an online statistical analysis tool [35,36].

## 3. Results

### 3.1. Transmission Electron Microscopy

**Figure 1:**
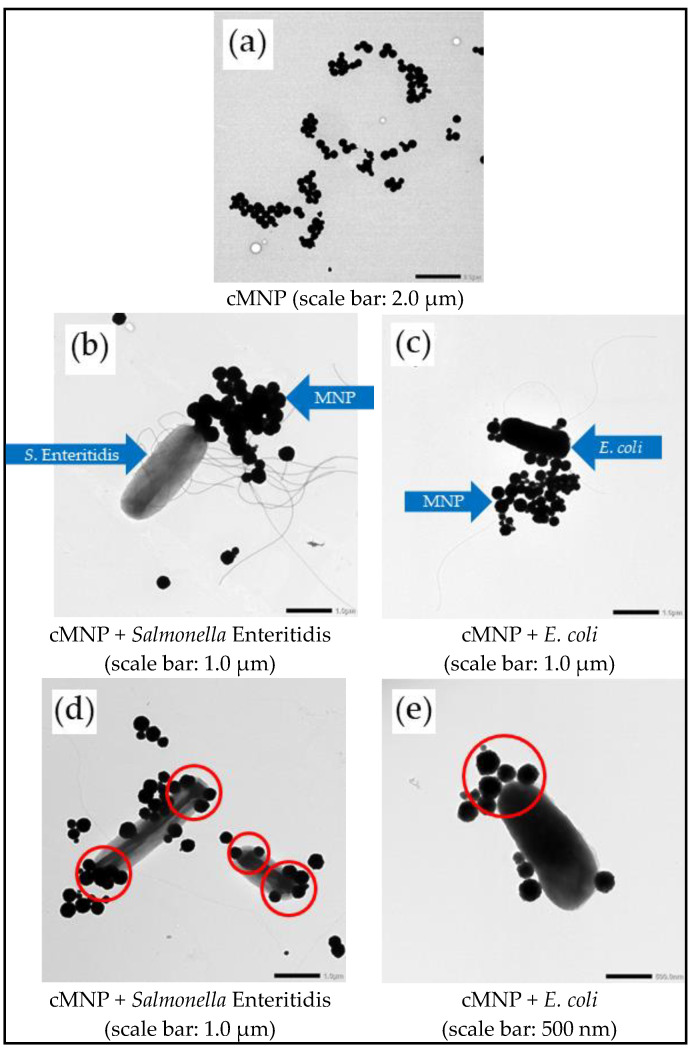
TEM images of the cMNP (**a**) and the conjugates formed with *Salmonella* Enteritidis and *E. coli*. The cMNP was bound to the flagella (**b**,**c**) and on the ends of the cells (**d**,**e**).

Figure 1 shows the TEM images of the cMNP and the conjugates formed with *Salmonella* Enteritidis and *E. coli*. The average size of the cMNP was determined to be 300 ± 82 nm with a saturation magnetization of 48.6 emu/g as interpolated from the magnetization curve Supplementary Material S1 [13]. A closer inspection of the TEM images of the cMNP–bacteria conjugates revealed that cMNP attached to the flagella of the bacteria (Figure 1b,c). It was also observed in multiple instances that the cMNP attached to the ends of *Salmonella* Enteritidis and *E. coli* rather than their sides (Figure 1d,e).

### 3.2. Adsorption Kinetics

**Figure 2:**
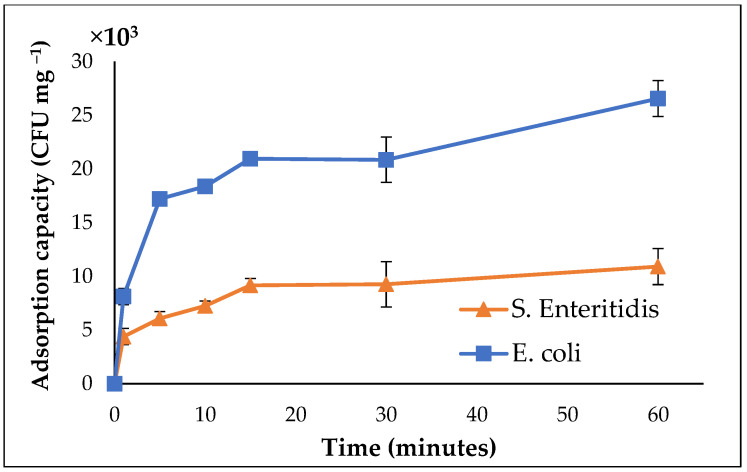
Kinetic plots of *Salmonella* Enteritidis and *E. coli* adsorption on MNPs (technical replicates = 3 for each point).

Batch adsorption studies investigated the interaction between *Salmonella* Enteritidis, *E. coli*, and cMNP. The adsorption kinetics were studied by determining the adsorption capacity of the cMNP at different incubation times. The kinetic plots of *Salmonella* Enteritidis and *E. coli* showed that the adsorption capacity for both bacteria started to level off after 15 min of incubation, suggesting that equilibrium was reached within 60 min (Figure 2). It was also observed that the adsorption capacity of the cMNP for *E. coli* was more significant than that for *Salmonella* Enteritidis across the different incubation periods.

The kinetic data were fitted to the Lagergren pseudo-first-order (PFO) and pseudo-second-order (PSO) kinetic models (Table 2) to estimate kinetic parameters. The PFO and PSO models, particularly their integrated form proposed by Ho and McKay [37], were two of the most commonly used adsorption kinetic models in the past two decades [25]. These two models were also used to model the kinetics of bacterial adsorption on various materials [25,27,31,38,39,40]. Using the linearized form is more convenient since it does not require complex calculations; however, several researchers have pointed out the disadvantages of using the linearized form when estimating kinetic parameters [25,27,32,41]. In this study, non-linear curve fitting was performed on both kinetics and isotherm experiments. Table 3 summarizes the results of curve fitting on the kinetic models.

Researchers used various quantitative parameters to validate how well the kinetic models fit the observed data. Several review articles provide a comprehensive summary of these quantitative parameters [25,27,42]. This study used the coefficient of determination (R^2^) and range normalized root mean square error (nRMSE). The adsorption data of *Salmonella* Enteritidis and *E. coli* on MNP was determined to fit PSO better than PFO (Table 3). It was also determined that the adsorption capacity measured at 60 min for *Salmonella* Enteritidis and *E. coli* agrees with the calculated equilibrium adsorption capacity, q_e_, from the PSO model, further validating the fit to the PSO model.

### 3.3. Adsorption Isotherms

Since the adsorption capacity at 60 min agrees with the calculated equilibrium adsorption capacity using the PSO, equilibrium experiments were performed with an incubation time of 60 min, assuming the equilibrium was achieved at 60 min. The adsorption capacity at 60 min was plotted against the equilibrium bacterial concentration on the supernatant and fitted to the Langmuir and Freundlich isotherms. Like in the case of curve fitting in linearized adsorption kinetic models, discrepancies between the experimental data and the predicted values were reported when using the linearized form of adsorption isotherms [32]; hence, this study used the non-linear form of the isotherms. The curve-fitting results (Table 4) revealed that the adsorption data fit both isotherms based on their R^2^ and nRMSE values.

Bacterial adsorption processes that follow the Langmuir and Freundlich isotherms have previously been reported (Table 5). However, it is advisable to look at the assumptions of the models to see which is more appropriate to describe the adsorption process. The Langmuir isotherm is an empirical model that assumes monolayer adsorption and that all binding sites are energetically homogeneous [32]. On the other hand, the Freundlich isotherm describes a non-ideal adsorption process and is not restricted to monolayer adsorption. It also assumes the heterogeneity of the surface wherein energetically favored binding sites are occupied first [32]. The heterogeneity of the cMNP surface can be attributed to the hydroxyl, amine, and acetylglucosamine functional groups from the chitosan coating and the exposed hydroxyl groups from the iron oxide core. In addition, the presence of various structures and functional groups that behave differently chemically contributes to the heterogeneity of the bacterial surface [43]. Based on the assumptions of the two isotherm models [32], the Freundlich isotherm is more appropriate than the Langmuir isotherm in describing the adsorption of the *Salmonella* Enteritidis and *E. coli* species on cMNP.

### 3.4. PZC and Zeta Potential Measurements

**Figure 3:**
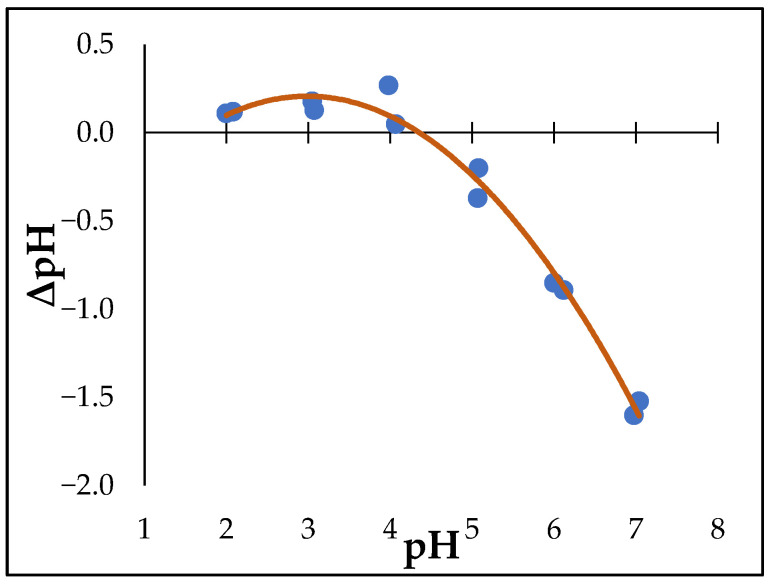
Point of zero charge determination plot for the MNP. The plot intersects the ΔpH = 0 axis at an approximately pH of 4.4 (technical replicates = 2).

The point of zero charge (PZC) of the cMNP was measured to determine the pH at which the cMNP surface would be net positively charged (Figure 3). The point of zero charge of the cMNP was determined at approximately pH 4.4, implying that the cMNP would have a net negatively charged surface at higher pH values. This is consistent with the PZC values of chitosan composites reported in previous studies (Table 6).

**Figure 4:**
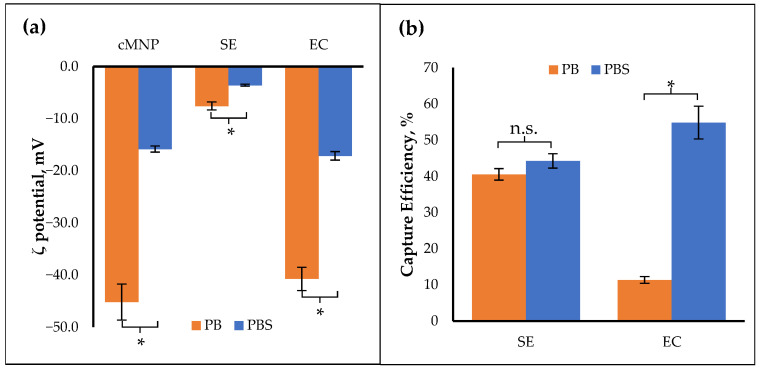
(**a**) Zeta potential of MNP, *Salmonella* Enteritidis (SE), and *E. coli* (EC) in phosphate buffer (PB) and phosphate-buffered saline (PBS). (**b**) Capture efficiency of *Salmonella* Enteritidis, and *E. coli* in phosphate buffer and phosphate-buffered saline. Asterisks “*” indicate the significant difference, and “n.s.” indicates the insignificant difference between treatments following Welch’s test (α = 0.05, technical replicates = 3).

The zeta potential of the bacteria provides insight into their surface charge. The zeta potential of the cMNP was negative, consistent with its PZC being less than the pH of PBS (pH = 7.4). The zeta potential of both bacteria was negative (Figure 4a). Bacteria tend to have a net negatively charged surface in near-neutral pH, with only a few species exhibiting a net positive charge [47,48]. For gram-negative bacteria like *Salmonella* and *E. coli*, the negative surface charge can be attributed to phosphoryl and carboxylate groups on the lipopolysaccharide on the outer membrane [49]. Despite having negative charges, the cMNP interacted significantly with both bacteria, as observed in the adsorption experiments performed in PBS. Bacterial cells have been shown to adsorb on materials with negatively charged surfaces [50,51]. This suggests that other types of interactions, such as hydrophobic and ligand–receptor interactions, also play a role in the adsorption process besides electrostatic interaction [43].

### 3.5. Effect of Ionic Strength

The effect of ionic strength on bacterial adsorption was investigated by performing adsorption experiments at different ionic strengths. Phosphate buffer has lower ionic strength than phosphate-buffered saline. The cMNP and the bacteria had significantly lower zeta potential magnitudes (*p* < 0.05) when the ionic strength of the solution was high (Figure 4a), consistent with prior studies that showed the inverse relationship between the zeta potential of particles and the ionic strength of the solution [52,53,54,55,56]. It was also determined that the effect of ionic strength on capture efficiency was more pronounced in *E. coli* (*p* = 0.004) than in *Salmonella* Enteritidis (*p* = 0.131) (Figure 4b).

### 3.6. Effect of pH

**Figure 5:**
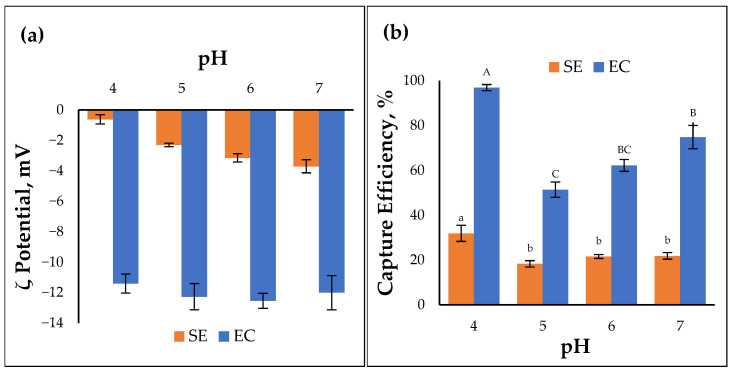
(**a**) Zeta potential of *Salmonella* Enteritidis (SE) and *E. coli* (EC) measured at different pH (technical replicates = 3). (**b**) Capture efficiency of *Salmonella* Enteritidis (left) and *E. coli* (right) measured at different pH. Similar letter labels above the bars indicate the non-significant difference between the treatments following Tukey’s HSD test (α = 0.05, technical replicates = 3).

Adsorption experiments were performed at various pH levels since the surface charge of the cMNP and the bacteria could be influenced by the pH of the solution. Both bacteria had negative zeta potentials from pH 4 to 7 (Figure 5a). The capture efficiency increased significantly at pH 4 compared to pH 5 to 7 (*p* < 0.05) (Figure 5b).

## 4. Discussion

The interaction between bacteria and surfaces can be viewed as a ligand–receptor interaction [43,48]. The binding of cMNP on flagella is consistent with earlier studies involving the adsorption of flagella-containing species like *Vibrio* spp. and *Pseudomonas fluorescens,* suggesting that the adsorption is not limited to the ends or tips but also includes other surfaces of these appendages [57,58,59]. The attachment of the MNP to the bacterial ends and flagella suggests that receptors binding to the cMNP can be present in these regions. This spatial limitation is characteristic of ligand–receptor interaction [43], where the protein receptors can be present in specific areas of the bacterial surface, corroborating the earlier proposed mechanism [20].

Adsorption studies have been performed to gain insight into the interaction between bacteria and various materials. The heterogeneity of the surface oil palm kernel biochar was determined to be an important factor in the adsorption of *Bacillus cereus*, *Bacillus salmalaya,* and *Bacillus amyloliquefaciens* based on the significant fit of the adsorption data on the Freundlich isotherm [28,29]. Fitting adsorption data on adsorption isotherms was performed to determine the effect of pH on the adsorption of *Pseudomonas putida* on nanostructured silicon carbide [30]. The maximum sorption capacity of different Fe-N-S-co-doped porous carbon materials for *E. coli* and *Bacillus cereus* was determined based on the obtained Langmuir isotherm parameters [60]. The diffusivities of *E. coli* and *Staphylococcus aureus* on single-walled carbon nanotubes were determined based on adsorption kinetic studies [61]. In this study, adsorption data were fitted on PFO and PSO kinetic models and Langmuir and Freundlich isotherms.

Adsorption experiments were performed using diluted bacterial cultures grown in TSB for 3–4 h. Past studies on *Salmonella* and *E. coli* growth kinetics show that these bacteria are in their logarithmic growth phase within 3–4 h [62,63,64]. These cultures were used to ensure uniform bacterial activity during the adsorption experiments. At the 10^3^–10^5^ CFU/mL concentration range, adsorption can be described using PSO kinetics and the Freundlich isotherm. This suggests that chemical sorption plays a role in *Salmonella* Enteritidis and *E. coli* adsorption on the cMNP. Bacterial adsorption that follows PSO kinetics [2,29,38] and the Freundlich isotherm [28,29,45] has been previously reported. Processes that follow PSO kinetics typically have chemical sorption as the rate-limiting step [37,65,66]. Examining the Freundlich parameters, both *Salmonella* Enteritidis and *E. coli* have a 1/*n* < 1, suggesting that the adsorption of these bacteria on the cMNP is favorable [32] and that chemical sorption is involved in the process [65]. Some bacterial appendages can specifically bind with certain chemical moieties on the material surface [43]. The F-17 fimbriae present in *E. coli* [67] bind with *N*-acetylglucosamine [68], a structural subunit of the chitosan coating of the cMNP. *Salmonella* has a giant non-fimbrial adhesin, SiiE, present on its surface that binds with *N*-acetylglucosamine [69].

The increase in the capture efficiency with increasing ionic strength could be attributed to the effect of ionic strength on the thickness of the electrical double layer of the cMNP and the bacteria. The thickness of the electrical double layer increases exponentially with decreasing ionic strength [70], resulting in a greater surface potential [70], as indicated by the zeta potential values. Since the cMNP is negatively charged in PB or PBS, the bacteria still experience repulsion. However, with a compressed electrical double layer at higher ionic strength, the distance between the cMNP and the bacteria is smaller. Filamentous bacterial structures and surface polymers can bridge this gap, overcoming the repulsion and thus binding with the cMNP [71,72]. This is consistent with earlier reports indicating the positive relationship between bacterial adsorption and ionic strength. Gram-negative cocci isolated from an unconfined aquifer were found to adsorb less on prepared sand samples when the ionic strength of the diluent was decreased tenfold [73]. The affinity of *Vibrio alginolyticus* to hydroxyapatite was found to increase with increasing ionic strength [74]. In addition, if the ionic strength is high enough that the repulsion will be short-ranged, hydrophobic interaction may bypass the repulsive forces [72]. Based on this information, it is possible that the difference in the influence of ionic strength on the capture efficiencies of *Salmonella* Enteritidis and *E. coli* could be attributed to the ability of the surface structures of *E. coli* to bridge its gap with cMNP. Lowering the pH to 4 (lower than the PZC of the MNP) results in the development of a positive charge on the surface of the MNP, allowing the negatively charged bacteria to interact strongly with the cMNP, thus resulting in higher capture efficiency.

The adsorption capacity for *Salmonella* Enteritidis is less than that for *E. coli,* suggesting that more MNP needs to interact with *Salmonella* Enteritidis to capture it than *E. coli*. This is consistent with the lower capture efficiency of *Salmonella* Enteritidis. *Salmonella* cells are, on average, larger than *E. coli* cells [75,76]. Due to its larger size, more cMNP interacts with it than *E. coli*, resulting in a lower adsorption capacity. The differences in the surface moieties and structures also contribute to the variation in capture efficiency. The SiiE protein of *Salmonella* may require the cMNP to be at a much closer distance for binding. In comparison, the F-17 fimbriae of *E. coli* can reach a longer distance to bind with cMNP, resulting in less efficient binding for *Salmonella* Enteritidis than *E. coli*.

In this study, adsorption experiments were performed at bacterial concentrations approaching the infectious dose of foodborne pathogens [77,78], complementing previous adsorption studies at higher cell concentrations (Table 5). This emphasizes the value of the results since adsorption studies are seldom performed at this concentration range. Understanding how the cMNP and pathogenic bacteria interact at this concentration range is vital in developing a rapid analysis sample preparation procedure based on these interactions. It should be noted that the results apply within the concentration range of 10^3^ and 10^5^ CFU/mL. As cell concentration may influence how the bacteria and the cMNP interact, validating this study at concentrations beyond the range is therefore recommended.

While many physicochemical forces are involved in bacterial adsorption, they are not independent [71], as they are all necessary to achieve adsorption. Planktonic bacteria prefer growing on surfaces rather than in the surrounding liquid. The cMNP serves as a surface where bacteria can latch on. The bacteria are transported near the surface by non-specific, long-range interactions such as Brownian motion and van der Waals attraction forces. Once they are near the surface, the influence of short-range interactions such as hydrogen bonding, ionic and dipole interactions, and hydrophobic interactions becomes more significant [79]. These forces constitute the first level of interaction between the bacteria and the surface [43]. Mathematical models such as the Derjaguin–Landa–Verwey–Overbeek (DLVO) and the extended DLVO theories are developed to describe these interactions in the context of colloidal systems [43,48]. However, the complexity of bacterial surfaces makes it difficult for these kinds of theoretical models to describe the interaction process adequately. Protruding filamentous structures on the surface of the bacteria, such as the F-17 fimbriae identified in *E. coli* and some *Salmonella* serotypes, can bridge the gap between the bacterial surface and the material’s surface [67]. On the other hand, the SiiE adhesin makes the *Salmonella* surface more complex [80].

This study focused on *Salmonella* Enteritidis and *E. coli* adsorption on cMNP in a controlled environment using buffers. Future research should aim to validate these findings in more complex matrices. Additionally, it would be beneficial to test other serotypes of *Salmonella* enterica and *E. coli* to see if similar patterns could be observed. Given the differences in cell morphology and surface structures between the species studied, it is also recommended to examine other significant genera, such as *Staphylococcus*, *Listeria*, and *Campylobacter*. Another limitation of this study is that the insights regarding the mechanism are derived from observations made on transmission electron microscopy images and adsorption experiments. Future studies that can provide direct evidence of specific receptors on the preferred binding sites are recommended. The application of the models is limited within the cell concentration range tested. Additional studies can be performed outside the range, especially in low contamination levels.

## 5. Conclusions

This study highlights the use of adsorption studies to investigate the interaction between *Salmonella* Enteritidis, *E. coli*, and cMNP at lower concentration ranges (10^3^–10^5^ CFU/mL). The results indicate that chemisorption (e.g., ligand–receptor, electrostatic, and hydrophobic interactions) is a major factor in the adsorption and that efficient cell capture can be obtained within at least 15 min of incubation. In addition, the ionic strength and pH of the sample play an important role in improving the performance of cMNP-based sample preparation. Future directions of this study include optimizing and validating *Salmonella* Enteritidis and *E. coli* adsorption from complex samples representative of matrices from food production and processing systems. Furthermore, the adsorption of other foodborne pathogens on the cMNP and the incorporation of the optimized MNP-based sample preparation on rapid detection platforms, such as nano-biosensor-based detection, will be explored.

## Figures and Tables

**Table 1:** Adsorption kinetic models used in this study.

Model	Function (Non-Linear Form) [25,26,27]
Pseudo-first-order (PFO)	$q_{t}=q_{e}\left(1-e^{-k_{1}t}\right)$
Pseudo-second-order (PSO)	$q_{t}=\frac{k_{2}{q_{e}}^{2}t}{1+k_{2}q_{e}t}$

*t*—time; *q_t_*—adsorption capacity at time *t*; and *q_e_*—adsorption capacity at equilibrium.

**Table 2:** Adsorption isotherms used in this study.

Model	Function (Non-Linear Form) [32,33]
Langmuir	$q_{e}=\frac{q_{m}K_{L}C_{e}}{1+K_{L}C_{e}}$
Freundlich	$q_{e}=K_{F}{C_{e}}^{\frac{1}{n}}$

*q_e_*—adsorption capacity at equilibrium; *C_e_*—equilibrium concentration of the supernatant; *q_m_*—maximum adsorption capacity; *K_L_*—Langmuir constant; *K_F_*—Freundlich constant; and 1/*n*—adsorption intensity.

**Table 3:** PFO and PSO model parameters determined for *Salmonella* Enteritidis and *E. coli* adsorption on chitosan-coated cMNP.

	*Salmonella* Enteritidis	*E. coli*
Pseudo-first-order (PFO)		
*k_1_*, t^−1^	0.215	0.311
*q_e_*, CFU mg^−1^	9.63 × 10^3^	2.21 × 10^4^
R^2^	0.879	0.928
nRMSE	0.109	0.084
Pseudo-second-order (PSO)		
*k_2_*, mg CFU^−1^ t^−1^	3.91 × 10^−5^	1.67 × 10^−5^
*q_e_*, CFU mg^−1^	1.03 × 10^4^	2.47 × 10^4^
R^2^	0.939	0.968
nRMSE	0.077	0.056

**Table 4:** Summary of curve fitting results on Langmuir and Freundlich isotherms.

	*Salmonella* Enteritidis	*E. coli*
Langmuir		
*K_L_*	2.23 × 10^−6^	6.93 × 10^−6^
*q_m_*	2.52 × 10^5^	7.96 × 10^5^
R^2^	0.999	0.968
nRMSE	0.010	0.061
Freundlich		
*K_F_*	1.49	33.0
*n*	1.11	1.24
R^2^	0.999	0.970
nRMSE	0.010	0.061

**Table 5:** Examples of experimental conditions considered by various researchers for batch adsorption studies.

Adsorbent	Bacterial Adsorbate	Bacterial Load, CFU mL^−1^	Adsorption Isotherm	Reference
Nanostructured silicon carbide	*Pseudomonas putida*	10^8^	Langmuir	[30]
Magnetic coated chitosan	*E. coli, Staphylococcus aureus*	10^8^	Langmuir	[12]
Pyrite	*Leptospirillum ferriphilum, Acidithiobacillus caldus*	10^7^	Langmuir	[44]
Biochar	*Bacillus cereus*	10^7^	Freundlich	[28]
Biochar	*Bacillus salmalaya, Bacillus amyloliquefaciens*	10^8^–10^9^	Freundlich	[29]
Soil	*E. coli, Salmonella sp.*	10^6^–10^9^	Freundlich	[45]
Chitosan-coated MNP	*E. coli, Salmonella* Enteritidis	10^3^–10^5^	Freundlich	This study

**Table 6:** PZC values of chitosan composites reported in previous studies.

Material	PZC Value	Reference
NH_2_-MIL-101(Al)/chitosan nanocomposite	4.6	[10]
Pectin–chitosan binary composites	3.8 (in DMSO) 4.7 (in water)	[46]
Chitosan/iron oxide nanocomposite	4 to 10	[11]
Chitosan-coated MNP	4.4	This study

## Data Availability

The data presented in this study are available upon request from the corresponding author.

## Supplementary Materials

The following supporting information can be downloaded at: https://www.mdpi.com/article/10.3390/cells14030225/s1, Reference to the magnetization curve; Equations to calculate adsorption capacity and capture efficiency.

## References

[1] Tripathy A., Nine M.J., Silva F.S. (2021). Biosensing Platform on Ferrite Magnetic Nanoparticles: Synthesis, Functionalization, Mechanism and Applications. Adv. Colloid Interface Sci..

[2] Darabdhara G., Boruah P.K., Hussain N., Borthakur P., Sharma B., Sengupta P., Das M.R. (2017). Magnetic Nanoparticles towards Efficient Adsorption of Gram Positive and Gram Negative Bacteria: An Investigation of Adsorption Parameters and Interaction Mechanism. Colloids Surf. Physicochem. Eng. Asp..

[3] Materón E.M., Miyazaki C.M., Carr O., Joshi N., Picciani P.H.S., Dalmaschio C.J., Davis F., Shimizu F.M. (2021). Magnetic Nanoparticles in Biomedical Applications: A Review. Appl. Surf. Sci. Adv..

[4] Kim S.-E., Tieu M.V., Hwang S.Y., Lee M.-H. (2020). Magnetic Particles: Their Applications from Sample Preparations to Biosensing Platforms. Micromachines.

[5] Silva S.M., Tavallaie R., Sandiford L., Tilley R.D., Justin Gooding J. (2016). Gold Coated Magnetic Nanoparticles: From Preparation to Surface Modification for Analytical and Biomedical Applications. Chem. Commun..

[6] Ha Y., Ko S., Kim I., Huang Y., Mohanty K., Huh C., Maynard J.A. (2018). Recent Advances Incorporating Superparamagnetic Nanoparticles into Immunoassays. ACS Appl. Nano Mater..

[7] Franco A.J.D.M., Merca F.E., Rodriguez M.S., Balidion J.F., Migo V.P., Amalin D.M., Alocilja E.C., Fernando L.M. (2019). DNA-Based Electrochemical Nanobiosensor for the Detection of Phytophthora Palmivora (Butler) Butler, Causing Black Pod Rot in Cacao (*Theobroma cacao* L.) Pods. Physiol. Mol. Plant Pathol..

[8] Pal S., Alocilja E.C. (2010). Electrically Active Magnetic Nanoparticles as Novel Concentrator and Electrochemical Redox Transducer in *Bacillus Anthracis* DNA Detection. Biosens. Bioelectron..

[9] Shaumbwa V.R., Liu D., Archer B., Li J., Su F. (2021). Preparation and Application of Magnetic Chitosan in Environmental Remediation and Other Fields: A Review. J. Appl. Polym. Sci..

[10] Azari A., Malakoutian M., Yaghmaeain K., Jaafarzadeh N., Shariatifar N., Mohammadi G., Masoudi M.R., Sadeghi R., Hamzeh S., Kamani H. (2022). Magnetic NH2-MIL-101(Al)/Chitosan Nanocomposite as a Novel Adsorbent for the Removal of Azithromycin: Modeling and Process Optimization. Sci. Rep..

[11] Keshvardoostchokami M., Piri F., Zamani A. (2017). One-Pot Synthesis of Chitosan/Iron Oxide Nanocomposite as an Eco-Friendly Bioadsorbent for Water Remediation of Methylene Blue. Micro Nano Lett..

[12] Shah K.H., Yameen M.A., Yousaf T., Waseem M., Fahad M., Sherazi T.A., Ahmad H. (2020). Adsorption of Bacteria by Highly Efficient, Economic and Biodegradable Magnetic Coated Chitosan Adsorbent. J. Solut. Chem..

[13] Matta L.L., Alocilja E.C. (2018). Carbohydrate Ligands on Magnetic Nanoparticles for Centrifuge-Free Extraction of Pathogenic Contaminants in Pasteurized Milk. J. Food Prot..

[14] Guarnieri A., Triunfo M., Scieuzo C., Ianniciello D., Tafi E., Hahn T., Zibek S., Salvia R., De Bonis A., Falabella P. (2022). Antimicrobial Properties of Chitosan from Different Developmental Stages of the Bioconverter Insect Hermetia Illucens. Sci. Rep..

[15] Aranaz I., Alcántara A.R., Civera M.C., Arias C., Elorza B., Heras Caballero A., Acosta N. (2021). Chitosan: An Overview of Its Properties and Applications. Polymers.

[16] Sharief S.A., Caliskan-Aydogan O., Alocilja E. (2023). Carbohydrate-Coated Magnetic and Gold Nanoparticles for Point-of-Use Food Contamination Testing. Biosens. Bioelectron. X.

[17] Sharief S.A., Caliskan-Aydogan O., Alocilja E.C. (2023). Carbohydrate-Coated Nanoparticles for PCR-Less Genomic Detection of *Salmonella* from Fresh Produce. Food Control.

[18] Caliskan-Aydogan O., Sharief S.A., Alocilja E.C. (2023). Rapid Isolation of Low-Level Carbapenem-Resistant *E. coli* from Water and Foods Using Glycan-Coated Magnetic Nanoparticles. Biosensors.

[19] Caliskan-Aydogan O., Sharief S.A., Alocilja E.C. (2023). Nanoparticle-Based Plasmonic Biosensor for the Unamplified Genomic Detection of Carbapenem-Resistant Bacteria. Diagnostics.

[20] Dester E., Alocilja E. (2022). Current Methods for Extraction and Concentration of Foodborne Bacteria with Glycan-Coated Magnetic Nanoparticles: A Review. Biosensors.

[21] Boodoo C., Dester E., Asadullah Sharief S., Alocilja E.C. (2023). Influence of Biological and Environmental Factors in the Extraction and Concentration of Foodborne Pathogens Using Glycan-Coated Magnetic Nanoparticles. J. Food Prot..

[22] Ghazy A., Nyarku R., Faraj R., Bentum K., Woube Y., Williams M., Alocilja E., Abebe W. (2024). Gold Nanoparticle-Based Plasmonic Detection of *Escherichia coli*, *Salmonella enterica*, *Campylobacter jejuni*, and *Listeria monocytogenes* from Bovine Fecal Samples. Microorganisms.

[23] Dester E., Kao K., Alocilja E.C. (2022). Detection of Unamplified *E. coli* O157 DNA Extracted from Large Food Samples Using a Gold Nanoparticle Colorimetric Biosensor. Biosensors.

[24] Bhusal N., Shrestha S., Pote N., Alocilja E. (2018). Nanoparticle-Based Biosensing of Tuberculosis, an Affordable and Practical Alternative to Current Methods. Biosensors.

[25] Revellame E.D., Fortela D.L., Sharp W., Hernandez R., Zappi M.E. (2020). Adsorption Kinetic Modeling Using Pseudo-First Order and Pseudo-Second Order Rate Laws: A Review. Clean. Eng. Technol..

[26] Largitte L., Pasquier R. (2016). A Review of the Kinetics Adsorption Models and Their Application to the Adsorption of Lead by an Activated Carbon. Chem. Eng. Res. Des..

[27] Simonin J.-P. (2016). On the Comparison of Pseudo-First Order and Pseudo-Second Order Rate Laws in the Modeling of Adsorption Kinetics. Chem. Eng. J..

[28] Ajeng A.A., Abdullah R., Junia A., Lau B.F., Ling T.C., Ismail S. (2021). Evaluation of Palm Kernel Shell Biochar for the Adsorption of Bacillus Cereus. Phys. Scr..

[29] Ajeng A.A., Abdullah R., Ling T.C., Ismail S. (2022). Adhesion of Bacillus Salmalaya and Bacillus Amyloliquefaciens on Oil Palm Kernel Shell Biochar: A Physicochemical Approach. J. Environ. Chem. Eng..

[30] Borkowski A., Szala M., Cłapa T. (2015). Adsorption Studies of the Gram-Negative Bacteria onto Nanostructured Silicon Carbide. Appl. Biochem. Biotechnol..

[31] Bwatanglang I.B., Magili S.T., Mohammad F., Al-Lohedan H.A., Soleiman A.A. (2023). Biomass-Based Silica/Calcium Carbonate Nanocomposites for the Adsorptive Removal of *Escherichia coli* from Aqueous Suspensions. Separations.

[32] Al-Ghouti M.A., Da’ana D.A. (2020). Guidelines for the Use and Interpretation of Adsorption Isotherm Models: A Review. J. Hazard. Mater..

[33] Wang J., Guo X. (2020). Adsorption Isotherm Models: Classification, Physical Meaning, Application and Solving Method. Chemosphere.

[34] Bakatula E.N., Richard D., Neculita C.M., Zagury G.J. (2018). Determination of Point of Zero Charge of Natural Organic Materials. Environ. Sci. Pollut. Res..

[35] Two-Sample t-Test Calculator (Welch’s t-Test). https://www.statskingdom.com/150MeanT2uneq.html.

[36] ANOVA Calculator—One Way ANOVA and Tukey HSD Test. https://www.statskingdom.com/180Anova1way.html.

[37] Ho Y.S., McKay G. (1999). Pseudo-Second Order Model for Sorption Processes. Process Biochem..

[38] Bwatanglang I.B., Mohammad F., Janet J.N., Dahan W.M., Al-Lohedan H.A., Soleiman A.A. (2023). Biosorption of *Escherichia coli* Using ZnO-Trimethyl Chitosan Nanocomposite Hydrogel Formed by the Green Synthesis Route. Gels.

[39] Ho Y.S., McKay G. (1998). A Comparison of Chemisorption Kinetic Models Applied to Pollutant Removal on Various Sorbents. Process Saf. Environ. Prot..

[40] Tan K.L., Hameed B.H. (2017). Insight into the Adsorption Kinetics Models for the Removal of Contaminants from Aqueous Solutions. J. Taiwan Inst. Chem. Eng..

[41] Ho Y.-S. (2006). Second-Order Kinetic Model for the Sorption of Cadmium onto Tree Fern: A Comparison of Linear and Non-Linear Methods. Water Res..

[42] Wang J., Guo X. (2020). Adsorption Kinetic Models: Physical Meanings, Applications, and Solving Methods. J. Hazard. Mater..

[43] Straub H., Bigger C.M., Valentin J., Abt D., Qin X.-H., Eberl L., Maniura-Weber K., Ren Q. (2019). Bacterial Adhesion on Soft Materials: Passive Physicochemical Interactions or Active Bacterial Mechanosensing?. Adv. Healthc. Mater..

[44] Song J., Lin J., Ren Y., Lin J. (2010). Competitive Adsorption of Binary Mixture of Leptospirillum Ferriphilum and Acidithiobacillus Caldus onto Pyrite. Biotechnol. Bioprocess Eng..

[45] Nola M., Njiné T., Boutin C., Servais P., Messouli M., Bidjeck L.M.N., Monkiedje A., Togouet S.H.Z., Kemka N. (2005). Sorption Kinetics of *Escherichia coli* and *Salmonella* sp. on Two Soil Layers Associated with a Groundwater Table in Yaounde, Cameroon (Central Africa). Int. J. Environ. Res. Public Health.

[46] Kong D., Wilson L.D. (2020). Uptake of Methylene Blue from Aqueous Solution by Pectin–Chitosan Binary Composites. J. Compos. Sci..

[47] Hermansson M. (1999). The DLVO Theory in Microbial Adhesion. Colloids Surf. B Biointerfaces.

[48] Jones G.W., Isaacson R.E. (1983). Proteinaceous Bacterial Adhesins and Their Receptors. Crit. Rev. Microbiol..

[49] Wilson W.W., Wade M.M., Holman S.C., Champlin F.R. (2001). Status of Methods for Assessing Bacterial Cell Surface Charge Properties Based on Zeta Potential Measurements. J. Microbiol. Methods.

[50] Oh J.K., Yegin Y., Yang F., Zhang M., Li J., Huang S., Verkhoturov S.V., Schweikert E.A., Perez-Lewis K., Scholar E.A. (2018). The Influence of Surface Chemistry on the Kinetics and Thermodynamics of Bacterial Adhesion. Sci. Rep..

[51] Zuki F.M., Edyvean R.G.J., Ali U.F.M., Pourzolfaghar H., Gafri H.F.S., Bzour M.I. (2022). The Impact of Ionic Strength and pH on the Interaction of *Pseudomonas Putida* to Minerals and Electrical Potential of Surfaces. Desalination Water Treat..

[52] Dong S., Zeng Z., Cai W., Zhou Z., Dou C., Liu H., Xia J. (2019). The Zeta Potentials of G-C3N4 Nanoparticles: Effect of Electrolyte, Ionic Strength, pH, and Humic Acid. J. Nanoparticle Res..

[53] Doukkali M., Patel R.B., Stepanov V., Hadim H. (2017). The Effect of Ionic Strength and pH on the Electrostatic Stabilization of NanoRDX. Propellants Explos. Pyrotech..

[54] Salgın S., Salgın U., Bahadır S. (2012). Zeta Potentials and Isoelectric Points of Biomolecules: The Effects of Ion Types and Ionic Strengths. Int. J. Electrochem. Sci..

[55] Hsu B.-M., Huang C. (2002). Influence of Ionic Strength and pH on Hydrophobicity and Zeta Potential of *Giardia* and *Cryptosporidium*. Colloids Surf. Physicochem. Eng. Asp..

[56] van der Mei H.C., Meinders J.M., Busscher H.J. (1994). The Influence of Ionic Strength and pH on Diffusion of Micro-Organisms with Different Structural Surface Features. Microbiology.

[57] Attridge S.R., Rowley D. (1983). The Role of the Flagellum in the Adherence of Vibrio Cholerae. J. Infect. Dis..

[58] Preston T.M., King C.A. (1984). Binding Sites for Bacterial Flagella at the Surface of the Soil Amoeba Acanthamoeba. Microbiology.

[59] Deflaun M.F., Tanzer A.S., McAteer A.L., Marshall B., Levy S.B. (1990). Development of an Adhesion Assay and Characterization of an Adhesion-Deficient Mutant of Pseudomonas Fluorescens. Appl. Environ. Microbiol..

[60] Borkowski A., Kiciński W., Szala M., Topolska J., Działak P., Syczewski M.D. (2020). Interactions of Fe–N–S Co-Doped Porous Carbons with Bacteria: Sorption Effect and Enzyme-Like Properties. Materials.

[61] Upadhyayula V.K.K., Deng S., Mitchell M.C., Smith G.B., Nair V.K., Ghoshroy S. (2008). Adsorption Kinetics of *Escherichia coli* and Staphylococcus Aureus on Single-Walled Carbon Nanotube Aggregates. Water Sci. Technol..

[62] Lee B.-S., Chen Y.-J., Wei T.-C., Ma T.-L., Chang C.-C. (2018). Comparison of Antibacterial Adhesion When Salivary Pellicle Is Coated on Both Poly(2-Hydroxyethyl-Methacrylate)- and Polyethylene-Glycol-Methacrylate-Grafted Poly(Methyl Methacrylate). Int. J. Mol. Sci..

[63] He H., Genovese K.J., Swaggerty C.L., Nisbet D.J., Kogut M.H. (2013). Nitric Oxide as a Biomarker of Intracellular *Salmonella* Viability and Identification of the Bacteriostatic Activity of Protein Kinase A Inhibitor H-89. PLoS ONE.

[64] Dhakal J., Sharma C.S., Nannapaneni R., McDaniel C.D., Kim T., Kiess A. (2019). Effect of Chlorine-Induced Sublethal Oxidative Stress on the Biofilm-Forming Ability of *Salmonella* at Different Temperatures, Nutrient Conditions, and Substrates. J. Food Prot..

[65] Sahoo T.R., Prelot B., Bonelli B., Freyria F.S., Rossetti I., Sethi R. (2020). Chapter 7—Adsorption Processes for the Removal of Contaminants from Wastewater: The Perspective Role of Nanomaterials and Nanotechnology. Nanomaterials for the Detection and Removal of Wastewater Pollutants.

[66] Bulut Y., Tez Z. (2007). Adsorption Studies on Ground Shells of Hazelnut and Almond. J. Hazard. Mater..

[67] Buts L., Bouckaert J., De Genst E., Loris R., Oscarson S., Lahmann M., Messens J., Brosens E., Wyns L., De Greve H. (2003). The Fimbrial Adhesin F17-G of Enterotoxigenic *Escherichia coli* Has an Immunoglobulin-like Lectin Domain That Binds N-Acetylglucosamine. Mol. Microbiol..

[68] Lewis A.L., Kohler J.J., Aebi M., Varki A., Cummings R.D., Esko J.D., Stanley P., Hart G.W., Aebi M., Mohnen D., Kinoshita T., Packer N.H., Prestegard J.H. (2022). Microbial Lectins: Hemagglutinins, Adhesins, and Toxins. Essentials of Glycobiology.

[69] Wagner C., Barlag B., Gerlach R.G., Deiwick J., Hensel M. (2014). The Almonella Enterica Giant Adhesin SiiE Binds to Polarized Epithelial Cells in a Lectin-like Manner. Cell. Microbiol..

[70] Balasubramanian K. (2010). Challenges in the Use of 1D Nanostructures for On-Chip Biosensing and Diagnostics: A Review. Biosens. Bioelectron..

[71] Daeschel M.A., McGuire J. (1998). Interrelationships between Protein Surface Adsorption and Bacterial Adhesion. Biotechnol. Genet. Eng. Rev..

[72] Poortinga A.T., Bos R., Norde W., Busscher H.J. (2002). Electric Double Layer Interactions in Bacterial Adhesion to Surfaces. Surf. Sci. Rep..

[73] Bolster C.H., Mills A.L., Hornberger G.M., Herman J.S. (2001). Effect of Surface Coatings, Grain Size, and Ionic Strength on the Maximum Attainable Coverage of Bacteria on Sand Surfaces. J. Contam. Hydrol..

[74] Gordon A.S., Millero F.J. (1984). Electrolyte Effects on Attachment of an Estuarine Bacterium. Appl. Environ. Microbiol..

[75] Andino A., Hanning I. (2015). *Salmonella enterica*: Survival, Colonization, and Virulence Differences among Serovars. Sci. World J..

[76] Shiomi D., Mori H., Niki H. (2009). Genetic Mechanism Regulating Bacterial Cell Shape and Metabolism. Commun. Integr. Biol..

[77] Tuttle J., Gomez T., Doyle M.P., Wells J.G., Zhao T., Tauxe R.V., Griffin P.M. (1999). Lessons from a Large Outbreak of *Escherichia coli* O157[Ratio]H7 Infections: Insights into the Infectious Dose and Method of Widespread Contamination of Hamburger Patties. Epidemiol. Infect..

[78] Mkangara M. (2023). Prevention and Control of Human *Salmonella enterica* Infections: An Implication in Food Safety. Int. J. Food Sci..

[79] An Y.H., Friedman R.J. (1998). Concise Review of Mechanisms of Bacterial Adhesion to Biomaterial Surfaces. J. Biomed. Mater. Res..

[80] Wagner C., Polke M., Gerlach R.G., Linke D., Stierhof Y.-D., Schwarz H., Hensel M. (2011). Functional Dissection of SiiE, a Giant Non-Fimbrial Adhesin of *Salmonella enterica*. Cell. Microbiol..

