# Supplementary material for: Adsorption Studies of Salmonella Enteritidis and Escherichia coli on Chitosan-Coated Magnetic Nanoparticles
Source: Cells. 2025 Feb 5;14(3):225. doi: 10.3390/cells14030225 (PMC11817960; doi:10.3390/cells14030225)
Supplement: Supplementary file 1 [file cells-14-00225-s001.zip › cells-3350947-supplementary.pdf]

Supplementary materials

S1. For the saturation magnetization, the magnetization curve can be found in:

Matta, L.L.; Alocilja, E.C. Carbohydrate Ligands on Magnetic Nanoparticles for Centrifuge-Free Extraction of Pathogenic Contaminants in Pasteurized Milk. *J. Food Prot.* **2018**, *81*, 1941–1949, doi:10.4315/0362-028X.JFP-18-040.

S2. Equations used in the calculation of adsorption capacity and capture efficiency:

Adsorption capacity:

$$q = \frac{\frac{Colony\ count\ from\ the\ residue, CFU}{Volume\ plated, \mu L} \times \frac{1000\ \mu L}{1\ mL} \times Resuspension\ volume, mL}{\frac{5\ mg\ MNP}{mL} \times Volume\ cMNP\ added, mL}$$

Capture Efficiency:

$$CE = \frac{Colony\ count\ from\ the\ supernatant, CFU}{Colony\ count\ from\ the\ residue, CFU + Colony\ count\ from\ the\ supernatant, CFU} \times 100$$
